# Pathology

**Published:** 1824-03-01

**Authors:** 


### Pathology.

### 1. *Observations on Inflammation of the Diaphragmatic Pleura.* By M. Andral, Jun.*

Whatever difference of opinion may exist respecting the possibility of distinguishing inflammation of the pleura from inflammation of the lungs, M. Andral is convinced that there are some symptoms which may generally evince the existence of that dangerous malady —*diaphragmatic pleuritis.* Boerhaave defines it thus:—"*Si morbus pleuritidi similis occupat eam membranæ pleuræ partem, quæ diaphragma ambit vel et ipsum septum medium, oritur morbus dirus quem paraphrenitidem appellant.*"—Aph. 907. It is evident that Boerhaave here confounds inflammation of the pleura with that of the muscular structure of the diaphragm. The symptoms assigned to the disease by Boerhaave are, acute fever; sharp pain in the region of the diaphragm, grievously augmented by inspiration, cough, sternutation, or attempts to vomit or pass fæces; short breathing, performed entirely by the ribs; depression of the hypochondria; risus sardonicus; delirium; convulsions. M. Andral now proceeds to relate cases of the disease observed in hospital practice.

*Case* 1. A man, 26 years of age, entered La Charité, in the month of April, 1822, having been seized, two days previously, with rigor followed by febrile heat. In the midst of this last, he felt an acute pain in the left hypochondrium along the margins of the false ribs, accompanied by much oppression. Hot fomention externally, and spirituous potations internally had no effect in dissipating this

---

* Archives Generales, October, 1823.

pain. He passed the night in great pain, and without sleep, having some hiccup. The succeeding day was also passed in pain, increased dyspnœa, frequent cough. On the *third day* he was seen by the reporter, who found him sitting up, and bent forward, with his hand constantly pressing on the hypochondrium, which was very tender to the touch. His countenance expressed the utmost anxiety, the inspirations were short, the cough frequent without any expectoration. No information could be gained by percussion or auscultation. The pulse was very quick and hard—the skin dry and burning. The intellectual and digestive functions were unimpaired. M. Lerminier suspected diaphragmatic pleuritis, and bled the patient to *twelve ounces*, after which twenty leeches were applied along the edges of the false ribs. In the course of the day, there was some diminution of the pain; but in the night it returned with increased intensity, accompanied by delirium. On the morning of the *fourth day*, the delirium subsided, but the pain and orthopnœa persisted—the pulse had lost nothing of its frequency or hardness—there were some transient convulsive movements of the muscles of the face. *Venesection to twelve ounces*—reapplication of twenty leeches to the side—diluents. Remission of the symptoms this day—an exacerbation of them all at night, with delirium. Sinapisms to the legs. *Fifth day.* Continuance of all the symptoms, with the addition of constant nausea. Blister to the neck. *Sixth day.* Great change in the countenance—lies on his back—voice extinct—hiccup. Died in the evening.

*Dissection.* The pleura covering the base of the left lung, and that opposite to it on the diaphragm was strongly injected, and coated with an albuminous exudation, filling up the space between the two portions of pleura. All other parts of this membrane and also the lungs were sound, as were the heart, pericardium, substance of the diaphragm, abdominal and cranial organs.

That this was a well marked case of uncomplicated diaphragmatic pleuritis, there can be no doubt. It affords a very fine example of the extensive influence which even a partial inflammation of a serous membrane exerts over the nervous system and various functions essential to life. Here, death was occasioned by the *functional* derangement of the circulation and respiration, induced by a very circumscribed phlogosis of the pleura. That life might have been preserved by any thing like active treatment, there is every probability. It is not a little surprising, that so late as 1822, M. Lerminier, a celebrated hospital physician of Paris, should allow a young man of 26 years of age to die of most acute pleuritis without abstracting more than 24 ounces of blood from the arm, at *two bleedings*, separated by a period of twenty-four hours!. It is evident that the patient should, in the first instance, have been bled to syncope, or relief of the pain—and that, instead of allowing the evening exacerbation to rise and ravage through the night unchecked, the patient should have been watched, and bled as the symptoms increased in violence. No purgatives, no antimonials were employed—in short, if M. Lerminier had wished to make his diagnosis turn out precisely what he had said, he could not have taken a more effectual method of accomplishing it than that which he pursued. The case affords an instructive lesson, however, to the junior practitioner.

*Case* 2. A tailor, 31 years of age, had for two years past been subject to repeated attacks of hæmoptysis, with habitual dry cough, and slight dyspnœa. On the 5th October, 1821, after getting wet with rain, he was seized with acute pain about the scrobiculus cordis, which obliged him to sit in a bent position forwards, and greatly incommoded his breathing. He applied a dozen of leeches to the part, and the pain subsided. Next day it recurred at intervals. On the 7th October, he was awakened, at three in the morning, by a violent pain stretching along the cartilages of the false ribs on the right side, and extending into the hypochondrium, of that side, accompanied by malaise, constant attempts to cough without power to do so freely, occasional vomitings. On entering the Charité, on the morning of the 8th October, the patient presented the following symptoms—pallor of the face, great anxiety in the countenance, short breathing performed by the ribs, constant short cough, pain at the place abovementioned, exasperated by pressure. Pressure on the epigastrium excited nausea and hiccup. The patient would not permit himself to be moved in the least degree—fever intense. Thirty leeches were applied to the right hypochondrium. On the 9th October, the pain excessive—venesection to *eight ounces*—twelve leeches to the epigastrium. In the course of the day, the pain in the epigastrium subsided, but that in the hypochondrium continued—breathing a little relieved by the bleeding. 10th October, an icteritious tint on the surface—in other respects the same. 11th. Pain relieved, breathing freer, cough not so urgent. In the evening exacerbation of the pain and all the other symptoms. Had bilious vomitings this night. 12th. Features contracted, some slight convulsive movements in the muscles of the face—orthopnœa—pulse quick and concentrated—skin hot—jaundice complete. Venesection to eight ounces. In the course of the day there was delirium. 13th. The pain not much complained of, except when coughing; but the breathing is greatly embarrassed, the pulse the same as before. The symptoms went on gradually increasing till the 21st October, when the patient died.

*Dissection.* The base of the right lung was separated from the diaphragm by a sero-purulent effusion, circumscribed in extent by membraniform concretions extending from the diaphragm to the lungs. This effusion had displaced both the lung and the liver. The other parts of the pleura were healthy—lungs interspersed with numerous miliary granulations—heart and pericardium sound, as were the abdominal viscera. The arachnoid membrane shewed signs of phlogosis, and there was some white serous effusion in the ventricles of the brain.

The same observations which we made on the first case will apply to this one. More active treatment might not only have prevented the sero-purulent effusion in the chest, but the supervention of the arachnitis, which was doubtless the ultimate cause of death.

The above are the only unequivocal cases of diaphragmatic pleuritis which our author has brought forward. The others are complicated with inflammation of the lungs or other portions of the pleura, and need not occupy our time here.

---

2. *Encysted Tumour.** A curious case lately occurred in the practice of Dr. Gregory. A woman applied at the dispensary, on the 3d of October, 1823, complaining of pain in the abdomen, quick pulse, foul tongue, frequent discharges of wind, great weakness of the legs and thighs. These complaints were of four months standing. In the lower part of the abdomen a firm swelling was discovered, but without fluctuation. On the 14th of the same month, Dr. G. visited this woman, and found the symptoms much aggravated, the pain being excruciating, and the abdomen much enlarged, tense and tender, with difficulty of breathing. Cathartics gave some relief, but on the 16th the symptoms all returned. Some fluctuation was now perceived, and on the 21st a trochar was pushed into the swelling, and about five pints of a transparent, brownish-coloured fluid were slowly drawn off. But it was evident that there was a large tumour within, untouched by the trocar. The relief obtained was trifling, and the poor woman was worn out with pain and irritative fever. She died on the 29th of October. The body was opened by Mr. Jeffreys next day, in presence of Dr. Gregory, Dr. James Johnson, and several other medical gentlemen, when the following appearances presented themselves.

"On dividing the abdominal parietes, a large firm tumor, of a black colour, was brought into view, attached by slight, and probably recently effused, bands of coagulable lymph, to the peritoneum lining the abdominal muscles. These adhesions were less firm towards the posterior part, so that, after separating the tumor from the parietes of the abdomen, it readily *turned out*, as the yolk of an egg separates from the white. This tumor occupied nearly the whole of the abdominal cavity from the ensiform cartilage to the pelvis, and was every where of a very dark colour, almost black. When cut into, it was found to contain a large quantity of a thick, fetid, chocolate-coloured fluid. The walls of the cyst were of unequal thickness: in many parts they were not less than two inches thick. Its external surface was *every where smooth*, making allowance for a loose covering of coagulable lymph. The internal surface of the coats of the cyst was rough and irregular. Its texture was throughout soft, spongy, and *rotten*; and, by many gentlemen present, was compared to that of a putrid placenta. Its whole weight, including that of the contained fluid, may probably have somewhat exceeded twelve pounds.

"The uterus and its appendages were now cut out, and carefully examined. Both ovaria were found in a perfectly healthy state; and the uterus itself was free from disease.

---

* Dr. Gregory. Med. and Phys. Journal, No. 298.

"The tumor being removed, gave us an opportunity of seeing that the peritoneum covering the bowels, liver, and other viscera, was every where of the same dark colour as the surface of the cyst; and it was in all parts covered with the same shreds of coagulable lymph as were witnessed in the tumor. The intestines were loosely glued together, and their coats appeared preternaturally thickened. The liver internally was of an ash-colour, and unhealthy texture. The stomach was not more than usually distended.

"The heart and lungs were free from all traces of disease."

We think there is great reason to believe that the above cyst was a solitary hydatid, whose parietes were, in several places, in a state of disease.

---

3. *Fever in the Bann and at Ascension.* Our readers are aware that we have always advocated the doctrine of "*contingent contagion*" in yellow fever—and indeed in most fevers. We have always stood aloof both from Dr. Bancroft on the one extreme, and Dr. Pym on the other; considering the exclusive doctrines of contagion and non-contagion as equally erroneous. That the yellow fever of the West Indies, and of countries whose temperature occasionally rises to the tropical degree, is rarely contagious, *under common circumstances of cleanliness and ventilation*, is as well ascertained as any fact in medicine. But that the same fever, under opposite circumstances of crowding, &c. may, and does, become contagious, is also proved by unquestionable evidence. The following case is that which has furnished Sir Gilbert Blane with materials for a letter to the Lords Commissioners of the Admiralty respecting the contagion of yellow fever. Early in the year 1823, the Bann sloop of war was much employed on the coast of Africa in searching for, and boarding, slave ships, many of whom were very sickly. A few days before the Bann sailed from the coast, she had free communication with the Caroline, timber ship, whose crew had been almost entirely swept off by fever. There was, also, an unprecedented mortality among the troops and settlers on the coast at this time. The Bann sailed from Sierra Leone in the latter end of March, 1823, and in a few days afterwards, a fever broke out, of a very malignant nature, wearing the general features of the endemic fevers of the West Indies and other similar climates. During the voyage to Ascension, of less than a month, the fever prevailed throughout the crew, and the sick were greatly crowded in consequence of the deluges of rain which fell on the passage. In this time thirteen men died, and twenty afterwards at Ascension. She landed her sick at the last-mentioned place, on the 25th of April, at which time the garrison was healthy. Some precautions, but not of a very rigid kind, were taken to prevent intercourse between the Bann's crew and the garrison; but notwithstanding this, the fever broke out about eight or ten days after the landing of the sick, and prevailed very generally through the small garrison on the island, of whom twenty-five died. We have seen all the documents respecting this unhappy occurrence, and can entertain no doubt of the contagious nature of the fever, and its communication to the garrison of Ascension. We have carefully perused the surgeon's journal of the Bann, and the only doubt in our minds is respecting the real nature and origin of the fever. The general features, as we before said, were those of the endemic fevers of hot climates; but the question is, whether a contagious fever might not have been carried from Malta, (whence the Caroline sailed for Sierra Leone,) and there, under a vertical sun, have assumed the garb of the fevers of the climate, with the power of propagating itself to the crew of the Bann, and so on? This, however, we only throw out as a query, professing our entire conviction that it was imported into Ascension through the medium of the Bann's crew. We are, indeed, more inclined to believe that the fever was originally produced by general causes on the coast of Africa, and acquired the dangerous property of contagion during the crowding of the sick on the passage to Ascension, from certain authentic intelligence which we have recently received from the West Indies. The account will probably be published, and, therefore, we shall no farther anticipate it than by stating a very few of the particulars. A British sloop of war lying in Antigua harbour began to show symptoms of the endemic fever of that unhealthy spot. She was, contrary to the surgeon's advice, hurried out to sea, and there the fever became general among the crew, evincing, at last, from the crowded state of the sick, a decidedly contagious character. They were obliged to land on a small desert island on the coast of America, and even *there* some strangers that visited the tents of the sick caught the fever. We mention this fact to show the impropriety of sending ships out to sea with a fever on board. Had the sick men of this sloop of war been landed at the hospital in Antigua, not a particle of contagion would have been evinced, and many lives would have been preserved. Finally, we may be permitted to reiterate our belief that contagion is an accidental adjunct to yellow fever, and by no means an essential part of its character. It is the child of circumstances.

---

4. *Hydrophobia.** It has been known, for a considerable time past, that Marochetti described pustules which he said rose under the tongues of people bitten by rabid animals, the opening of which, in conjunction with decoction of broom, he considered as a security against the disease. Little credit appears to have been given to this story in England; but, in the September number of the Journal General de Medecine we find an apparently partial confirmation of Marochetti's assertions, and we, therefore, deem it proper to notice the statement.

On the 12th of October, 1822, several people and some sheep were bitten by a rabid dog at Boulay and the vicinity. M. Magistel cauterized the wounds, and kept a look out for the pustules abovementioned. In several instances he did observe these sublingual pustules, some appearing on the 6th day from the bite, some later,

* M. Magistel. Journal General de Medecine, September 1823.

and the latest on the 32 day. He describes two species, the crystalline and opake—the *former* prominent, rounded, the size of a hemp-seed, being transparent, and containing a colourless aqueous fluid. The opake pustules, on the other hand, are flat, circular, and the size of a lentil, covered by a brownish pellicle. The first species appear on the inferior superficies of the tongue—the second imbedded, as it were, in the lingual substance. Both species are situated chiefly on the sides of the frenum linguæ. All those who were bitten exhibited the opake species of pustules. Not so with the crystalline. The cautery soon cured both species. The decoctum genistæ was carefully administered, and employed also as a lotion to the wounds. Of ten who were bitten, and attended by M. Magistel, five died hydrophobic. The prevention, therefore, of hydrophobia, by cauterizing the sublingual pustules is not confirmed. But it is a curious phenomenon, the appearance of the pustules in such a situation, and deserves to be further investigated, as it may ultimately lead to some insight into the nature of this dreadful disease.

5. *Delirium Ebriositatis.** Mr. Blake, while serving in the West Indies, where rum is cheap and deleterious, had opportunities of seeing much of the above complaint. He thinks that Pearson, Armstrong, Sutton, and others, who have written on delirium tremens, have not paid sufficient attention to the *stages*, phenomena, and some of the pathological characters of the malady. Mr. B. divides it into three distinct stages. In ten cases brought forward by our author, the first stage, or that of nervous exhaustion, came on within five days from the admission into hospital, or, in other words, the abstinence from spirituous liquors. The *first stage* was evinced by coldness of the hands and feet—debility, and diminution of temperature—nausea and vomiting—nervous tremors of the hands and tongue—dejection of spirits—oppression at the præcordia—interrupted slumbers—frightful dreams.

As the second stage approached, the countenance became wild—the patient became restless, and quick in his answers, with an anxiety to perform immediately whatever was desired. This stage rarely lasts more than a few hours. This stage once established, a high state of nervous irritation follows. Mental alienation—small quick pulse—increase of heat on the surface—clammy sweats—presentation of frightful forms to the imagination—obstinate pervigilium. When these symptoms have continued one, two, or three days, the solution, if not by death, generally takes place in a strong tendency to sleep, which, as soon as it supervenes, lasts from six to eighteen hours—constituting what our author considers the third stage of the disease—"or general relaxation of the nervous action"—to which, convalescence, in his experience, always succeeded. But where this third or sleeping stage did not take place, the general symptoms increased in violence—the mental irritation became more severe—the patient made

* Mr. Blake. Ed. Journal, No. 78.

excessive struggles, attended with copious perspiration, at last of a deadly coldness—rapid pulse, and thready—tremor of the whole frame approaching to subsultus tendinum—contracted pupils—pale countenance—brown and dry tongue—incessant muttering. During this distressing scene the patient complains of no local pain; and if asked "how he does?" replies "very well."

The following rationale of the exciting cause is chaos itfelf. "The *exciting* cause appears to me to be the sudden cessation of the application of such stimuli to the nervous system, (through the medium of the digestive organs,) the powers of which sink to the lowest ebb; and in endeavouring to rally, and establish the equilibrium between the nervous and vascular systems, its efforts exceed the debilitated faculties of the sensorium, and thus excite delirium." If Mr. Blake comprehended his own meaning it is more than we can do.

When this disease terminates fatally, it does not appear to our author to be owing to venous congestion. He would rather ascribe it to serous effusion. He gives, however, but one dissection in favour of his pathological doctrine. The greater number of those cases which we have opened and seen opened, presented little or no effusion. Some presented turgescence of vessels; but others a pallor and collapse of the whole vascular system of the brain.

Our author's methodus medendi varies with the stage of the complaint. If we see the patient early before delirium sets in, and where gastric derangement is present, he advises effervescing draughts with ten drops of laudanum every two hours. In the intermediate hours he has been in the habit of giving an ounce of rum with warm water and sugar, together with the warm bath, or tepid or cold affusion according to the circumstances of the case. He permits soup, arrow-root, sago, or other mild nutriment, to be taken in moderate quantities. When constipation prevailed, a drop of croton oil was found a useful medicine. If these means fail in preventing the second stage or that of reaction, then he advises full doses of opium, diffusible stimuli, and antispasmodics. To these he has been in the habit of adding calomel and Dover's powder, two grains of the former to six of the latter, every two hours, until the system became affected, or the disease yielded. Moral management is properly insisted on; and upon the whole we are inclined to commend Mr. Blake's paper, notwithstanding the confusion of ideas which seems to prevail with our author when attempting an explanation of the morbid phenomena.

---

6. *Scirrhous Cæcum mistaken for Aneurism.** On the 13th of February, 1822, Dr. Beezeley was called to visit a young farmer, 22 years of age, confined to bed with a severe attack of "palpitatio cordis," his pulse beating at the rate of 110 in the minute—his countenance pale, voice weak, and respiration somewhat impeded, skin moist, head-ache, bowels costive, occasional sickness at stomach,

---

* Philadelphia Journal, No. 12, August 1823.

and soreness in the chest, occasioned by the violent palpitation which had lasted about 36 hours. On examination, Dr. B. found a hard and irregular tumour in the right iliac region, "seeming to be made up of two, united by a line running parallel with the linea alba, and extended from Poupart's ligament, to about an inch above a line drawn from one anterior superior spinous process to the other, and from near the ilium to the linea alba, where its outline suddenly receded towards the spine." There was no perceptible pulsation at this time, nor for several weeks aftewards. He attributed the tumour to a violent blow from a plough, about eight months previously to the appearance of the tumour. Three months after receiving the blow, he began to be attacked with colic, constipation, and dyspeptic symptoms, for which various remedies were prescribed without success. In progress of time, several physicians were consulted, and as pulsation now became a feature of the disease, it was supposed to be aneurism of the external iliac artery. This belief was strengthened afterwards by another tumour, the size of an orange, springing up directly over the aorta, above the umbilicus, which pulsated so strongly, as to be perceptible through the bed-clothes. He died in a state of the greatest emaciation, and the following minutes shewed that the doctors were all mistaken in the nature of the case.

*Dissection.* On opening the abdomen, a large tumour presented itself, stretching from the liver to the iliac region, and extending over the spine into the left. This tumour was found to consist of four inches of the ileum, the whole of the cæcum, and five inches of the colon, in a scirrhous state, the mesenteric glands being, also, greatly enlarged and indurated. There were ulcerations in the interior of these scirrhous intestines, the coats of which, in some places, were more than an inch in thickness.

---

7. *Sudden Death, with fallacious Symptoms.** The great benefit resulting from *post mortem* examinations, consists in correcting the fallacy of symptoms. For if, by these last *alone*, the nature and seats of disease could be ascertained, of what use would be the dirty work of dissection, as far as the physician is concerned? But the fact is, that symptomatology and dissection are, in medicine, what the log-line and lunar observations are, in navigation. Symptomatology is our daily business in the practice of physic, and, without it, we could do nothing. But, every now and then a *post mortem* examination rectifies erroneous judgments which we have made during the life of the patient; in the same way that a lunar observation, when it can be obtained, corrects the fallacies of the log-line, produced by currents and counter-currents, the operations of which were concealed from the mariner's view.

A young man, 24 years of age, of good health and constitution, complained, in the month of January, of slight giddiness in his head,

---

* Journal Général de Medecine, Vol. LXXIII. p. 303.—M. Gaugiran, M.D. of Toulouse.

with a sense of tightness or oppression about the chest, on taking a deep inspiration. His tongue was furred in the mornings, his appetite diminished, and his digestion somewhat depraved. Having gone into the country, he was there seized with vomiting, followed by profuse perspirations. The same thing recurred a few days afterwards. He now returned to Toulouse, still affected with nausea and occasional vomitings. M. Gaugiran put him on a rigid regimen and diluent drinks. On the 1st of February, the vomiting returned, not again to cease. Dr. G. now examined his patient carefully. The abdominal viscera appeared in a sound state. He had never, to his knowledge, received any blow or fall on the head. An antimonial emetic was prescribed on the old principle, "*vomitus vomitu curatur.*" The patient vomited a great deal of bile, and, also, a lumbricus of considerable length. The enemy was now thought to be dislodged; but the vomitings continued with more violence than ever, and neither food nor medicine could be retained on the stomach. Blisters to the epigastrium—opium—assafœtida—leeches to the anus—calomel, &c. were prescribed, but without effect. On the 8th of February, the day on which he died, and now for the first time, the patient complained of pain in the occipital region, which became, in the course of the day, so excruciating, that he could not bear to be turned in his bed. He expired in the evening of this day.

*Dissection.* Messrs. Delpech, Gaugiran, and Naudin, were present when the body was opened. The brain being exposed, four or five fungous vegetations were seen to spring from the upper part of the dura mater, and opposite to them, were seen corresponding fossæ or depressions in the parietal bones. On the anterior superior part of both hemispheres, there was a morbid adhesion of the dura mater and arachnoid. There was an incipient patch of ossification at the upper part of the falciform process. From six to seven ounces of serum were found in the left lateral ventricle, and between four and five ounces in the right. The sinuses and vessels of the brain were gorged, as if injected, with blood. The mucous membrane of the stomach was phlogosed in a few points; and the liver was gorged with blood, and of a deeper colour than natural.

Dr. Gaugiran is of opinion, that this young man must have received a blow or fall upon the head, at some former period, though he could not recollect the circumstance; and M. Burdin, in his report on the case, is of a similar opinion. We cannot see any reason why there must *necessarily* have been external violence, because there was internal derangement of structure. We believe, however, with M. Burdin, and, indeed, the present case offers an example, "*that an organic disease of the head may manifest itself during life* ONLY *by functional disorder of the stomach.*" At the same time, we apprehend that, in general, an accurate observation of symptoms, and a minute enquiry into the cerebral functions, in such cases, would lead to the detection of the seat of the evil. This case shews the necessity of examining the functions of *all* the great organs, in obscure and anomalous affections. We suspect that, such a quantity of water could not have been effused into the ventricles of the brain, in this case, without some corresponding symptoms—at all events, the doubtful precept—"*vomitus vomitu curatur*," might, in this instance, at least, be changed to—"*cephalitis vomitu augetur*." French therapeutics are, in general, experiments in pathology, and the specimens which they thus produce, are far superior to ours, where the ravages of disease are modified, if they cannot be arrested, by the power of medicine.

*P.S.* The following case, which we have observed in another number of the same journal, bears upon the case just related.

A man, near 70 years of age, who had enjoyed good health for some time before, became affected with periodical vomiting, which returned every day, and, sometimes, twice a day, without any assignable cause. This vomiting resisted every kind of treatment that was employed by the best physicians in Paris. He then applied to the charlatan tribe, but with no better success. The patient left Paris to return to his native place in the country, having now laboured under the complaint for many months. On his way down, the voiture was upset, and he received a severe and extensive wound in his head, from which a great quantity of blood flowed, not only at the time, but for many hours afterwards. The vomiting, from this time, entirely ceased.

Here, we think, there is ground to suppose the real origin of the gastric affection was situated in the head, and, it is pretty evident, that the bungling voiturier proved a better physician than any that could be found in Paris.

---

8. *Pneumo-Thorax.** We are sorry to see the increasing propensity of medical men, to publish their papers in the *Philosophical* Transactions, rather than in the medical transactions or medical journals of the country. They ought to consider that, in the Provinces, not one medical man in five hundred, ever reads the Philosophical Transactions—and that, even in the metropolis, not one in fifty does so. Nor do we think the abovementioned mode of publication adds, in the least, to the respectability of such papers. The Medico-Chirurgical Transactions, the quarterly, and the monthly Journals of this metropolis, are respectable enough for any communication that issues from the press. It is true that, through the medium of periodical journals, the professional contents of the Philosophical Transactions are pretty generally circulated through the world; but then, it is often in a very curtailed state. There are many subjects, too, of a pure medical nature, which are certainly not adapted for the non-professional readers of the Philosophical Transactions. Of this kind we deem the present paper of Dr. Davy.

A soldier, of middle age, came into the Military Hospital of Chatham, in the month of January, 1823, and died on the 11th of February. On proceeding to examine the body, it was observed, that the

* Dr. Davy. Philosophical Transactions, 1823.

right side of the chest was much more protuberant than the left, and on percussion emitted a hollow sound. When the abdomen was opened, the diaphragm was seen protruding into the right hypochondrium, in a convex instead of a concave form. By putting the body under water and then making an aperture into the right side of the chest, 212 cubic inches of air were collected, besides several that escaped. The inner surface of the pleura was covered with a thin layer of coagulable lymph—the lung of that side was greatly compressed, and on being inflated by a pair of bellows, the air freely passed into the cavity of the pleura on that side, through an ulcerated opening in the superior lobe, where a vomica was detected communicating with the trachea by a large bronchial tube. Various small tubercles, in different states of forwardness, were also found in the right lung.

The air extricated from the chest when chemically examined, was found to consist of 8 per cent carbonic acid gas, and 92 per cent of azotic gas. What then had become of the oxygen (supposing it to have been common atmospheric air when extravasated) and whence came the carbonic acid gas?—To determine the former, common air was injected into the pleura of a dog, and the opening closed. After a lapse of 48 hours the dog was killed, and eight cubic inches of air obtained, shewing slight traces of carbonic acid gas, and consisting of 93 per cent of oxygen, and 7 per cent of azotic gas. This experiment, Dr. D. thinks, appears to shew that oxygen was absorbed in a greater proportion than azote, in the case of the dog. In respect to the man, it may be supposed that the carbonic acid gas, as formed in the lungs, had escaped with atmospheric air into the pleura, where it was le[illegible] readily absorbed than oxygen. To ascertain this last position another experiment was made.

A bladder was filled (about 30 cubic inches) with air, consisting of atmospheric air 80 parts and carbonic acid gas 20 parts. At one end of the bladder was a stop-cock, and at the other a trocar. The latter was thrust through the integuments of the chest on the right side of a dog—the stilet was withdrawn into the bladder, and the air then rushed through the canula into the thorax, being partly forced back again on expiration; the quantity retained being about ten cubic inches. The canula was then withdrawn and the wound closed. The health of the animal appearing to suffer but little, the operation was repeated two days after, on the opposite side, the injected air being 75 of common air to 25 of carbonic acid gas. Twenty-four hours afterwards the dog was killed, not having appeared to suffer much from either operation. About three cubic inches only of air were found in the *right* side, consisting of 18 carbonic acid gas, 78 azotic gas, and 3 of oxygen gas. The air admitted had consisted of about 20 carbonic acid gas, 63 azotic, and 16 oxygen, shewing, apparently, that, during a three days' sojourn in the pleura, the oxygen had been absorbed in a greater degree than the carbonic acid gas, and the latter in a greater degree than the azote. Some other experiments were made, tending to shew that the carbonic acid gas was not a secretion from the pleura, but on the contrary, shewing that this membrane has the power or property of absorbing gases, exhibiting a preference for some over others.

We have given an abstract of the above case and experiments for the edification of chemico-physiological readers—the following will be perused with more interest by our pathological brethren.

*Case of Pneumo-thorax.* A soldier was sent home invalided from the West Indies for hæmoptysis which had succeeded a severe fall on the left side of the chest received about 18 months previously. He was admitted into the Military Hospital, Chatham, on the 9th May 1823. On the morning of the 13th, after a violent fit of coughing, symptoms of pneumo-thorax came on suddenly, and continued increasing till the 21st. The most prominent of these symptoms were, sensation of extreme tightness about the chest and abdomen—rapid and difficult inspirations, between 30 and 40 in the minute—great anxiety and agitation of mind—pulse 130—cold sweats—prostration of strength. On examining thc chest, the left side was found to be more prominent than the other, and larger in all its dimensions. It was tense, and, on percussion, sounded remarkably hollow and tympanitic. The heart pulsated on the right side under the mamilla. It was determined to tap the chest. A trocar was attached to an empty bladder, and the parietes of the chest perforated between the 8th and 9th ribs. Only about five cubic inches of air rushed out, shewing that adhesions probably existed at this point. Next day another puncture was made below the left papilla, when a large quantity of air rushed out and distended the bladder. When the bladder was removed, air continued to rush from the chest for several seconds, as if from a blow-pipe. When this ceased the canula was removed, and the wound healed. The relief was great and sudden. The patient continued to improve up to the 17th June, on which day Dr. Davy's paper for the Royal Society was closed. At this period the patient's appetite was good— his cough but little troublesome—he could lie on the left side—the wounds were healed—and the chest considerably diminished in volume, as well as less tense and tympanitic. The heart, however, could still be felt on the right side, and the fluctuation of a fluid was perceptible in the left. Dr. D. entertained hopes of recovery.

The air which had rushed out into the bladder was found to consist of 93 parts of azotic gas, and 7 of carbonic acid gas, being almost the same as that found in the preceding fatal case.

We should be glad to know the final result of the second case, and hope the Doctor will favour the profession with an account of it.

---

9. *Blue disease.** Dr. Olivry, of Quimper, has lately related a case of this organic imperfection in the circulating system. A boy six years of age came under his notice, whose stature was not proportioned to his years, and whose cheeks, lips, hands, and arms,

---

* Dr. Olivry.—Journal Général de Medecine.

continued of a blue colour from birth. The pulse was irregular, small, and feeble; and the hand placed over the region of the heart felt irregular pulsations accompanied by a peculiar noise difficult to describe. The child was also subject to dyspnœa which sometimes threatened his life. He could not lie down horizontally, but preferred the sitting posture, and still more that of leaning forward. The natural functions, however, were regular; but he was very sensible to cold, and in the middle of summer his skin was like ice. His temper was violent and disagreeable; but his mental capacity was astonishingly precocious. He partook not of any of the pastimes of youth, but kept retired, and apparently in a melancholy mood. He loved to sleep, and when awakened was always in a violent passion, at which periods the blue colour became deeper in the parts above-mentioned, and even spread to the trunk of the body. Being seized with croup, he died in thirty hours.

*Dissection.* Traces of inflammation on the mucous membrane of the glottis, larynx, and trachea. The bronchiæ were filled with a whitish and thick fluid. The cavities of the pleura contained some serum—the lungs were turgid, and of a reddish and violet colour—and when cut into disgorged blood of a darker colour than natural. The size of the heart was natural. The foramen ovale was obliterated, as was also the canalis arteriosus. But there was a free communication between the two ventricles, "which permitted the blood to pass, during ventricular contraction, either into the aorta or pulmonary artery." This is not a very clear, or indeed a very correct statement. If the two ventricles act at the same time, the one would resist the regurgitation of blood from the other, in a great degree at least, and the blood would consequently take its natural course from both ventricles. But the circumstance of the septum ventriculorum being perforated, admitted of a certain degree of admixture of the black and red blood in the two main chambers of the heart—and this is the simple and true pathology of the case.

M. Corvisart relates a case of similar malformation, on which we shall have occaison to make a few remarks.

A boy 12½ years of age was admitted into the Clinique Interne on the 21st of April 1797, ill, according to the accounts of the parents, only five months; but from the violent palpitations one would be inclined to date the origin of the disease at a more remote period. The child's countenance was puffed—lips violet coloured—breathing remarkably embarrassed—action of the heart violent and irregular, accompanied by a peculiar noise. Yet the pulse, though small and weak, was regular. The little patient could not lie horizontally. While at the hospital the disease made progress, the attacks of suffocation becoming more and more distressing, and he died on the 25th of April.

*Dissection.* The size of the heart was considerably augmented in all the chambers, but *especially the right ventricle.* Between the two ventricles a foramen was found, capable of admitting the point of the little finger. At one side of this aperture of communication were seen two small tubercles of a red colour. One of the semilunar valves of the aorta was eroded, so as to permit a reflux of blood into the left ventricle during each diastole of the heart.

M. Corvisart seems to incline to the opinion that this communication between the two ventricles was not an original malformation, but that it had taken place in correspondence with the date of the symptoms. To this opinion we are not inclined to lean. That the symptoms did not shew themselves till five months before the patient's death, is no proof that the communication did not exist previously between the ventricles. So long as the two chambers act equally and naturally, very little inconvenience will be felt from an opening in the septum ventriculorum, because an equal pressure is made, and an equal resistance obtains in each side of the heart. But whenever one side of the heart acts with more strength than its due proportion, then blood will be thrown, at each contraction of that side, not only into the corresponding artery, but through the unnatural aperture into the other ventricle, thus producing a mixture of the black and red blood. This appears to have been the case in M. Corvisart's patient, for he distinctly states that the parietes of the right ventricle were thicker than natural, while those of the left were not increased in thickness.

---

10. *Paralysis.** Dr. Prichard endeavours to draw still more the attention of the faculty to affections of the spinal marrow, as producing or influencing diseases. His own attention was first attracted to the subject by a perusal of M. Esquirol's observations, and by the dissection of some cases of chorea. As dissections in this disease are rare, we shall briefly advert to Dr. Prichard's cases. In the *first*, a girl of 19 years died after an attack of acute rheumatism, and with the symptoms of chorea on her. Six drachms of serous fluid were found in the brain, and adhesion of the pericardium to the heart. In this case the spinal canal was not examined.

In the *second case*, a girl of seven years had been afflicted with chorea since her infancy. In the infirmary she became delirious and died. In the sheath of the medulla spinalis there was a great quantity of serous fluid; and the vessels on the surface of the spinal marrow were finely injected with blood. There was considerable effusion over and under the meninges of the brain.

The *third case* was also that of a young female, aged 19. She was admitted with chorea, and died about a month afterwards delirious, or rather maniacal. There was no striking or remarkable disease of the encephalon; but there was rather more than natural of serum under the membranes. On raising the trunk and depressing the head, between one and two ounces of serous fluid issued from the cavity of the theca vertebralis. On opening the spinal canal the membranes were found in a highly injected state.

The *fourth case* was a girl of 14 years, who had been seized after

---

* Dr. Prichard. Med. Repos. No. 1, New Series.

a fright with tremor and general agitation, which gradually assumed the character of chorea, and continued till the last moments of her life. On dissection, when the theca vertebralis was slit open, about an ounce of serous fluid escaped—the vessels of the medulla were distended—and at one part there was a layer of coagulable lymph. The brain was unusually red, and its substance vascular.

From the above dissections and the observations of M. Esquirol, our author is inclined to place the pathology of chorea and also of epilepsy and other disorders chiefly affecting the functions of the muscular system, in the medulla spinalis. "Morbid causes, says our author, acting within the spinal canal are likely to produce effects similar to those which arise from injuries or a diseased state of the vertebral column. In those instances where we find paralysis supervening on chorea or epilepsy, particularly when the paralytic state comes on gradually, we may often expect to find the cause of the disease in the spinal marrow. The same observation may be applied to paraplegia;—and in hemiplegia, at least in cases of palsy affecting an upper or lower extremity, the morbid cause may be in the spine." Several cases are related in support of the above observations, but for these we must refer to the original communication. The object of Dr. Prichard, in this paper, is to recommend blisters and issues to the spine, in paralytic affections—*after* the necessary depletive measures have been pursued. As an internal medicine he strongly praises the effects of the oil of turpentine, in doses of one to two drachms three times a day. The communication closes with a recommendation to establish drains in the neighbourhood of the spine and head in some other nervous diseases, as epilepsy, &c.

---

11. *Cholera Epidemica.** Dr. Robson, was a passenger in His Majesty's ship Malabar from the coast of India to the Equator. The The ship's crew began to be affected early in April 1819, and in a few days, 89 went through the disease, of whom 14 died—a smaller mortality, Dr. R. observes, than in any of the reports he has seen. It is not so small a proportion, however, as is recorded in some of the reports. Dr. R. pronounces that venesection would not have succeeded in a single instance, although it was never tried but in two cases. Small doses of calomel and opium not being found to answer, they had recourse to a combination in the proportion of one grain of opium to a scruple of calomel for a dose. This practice proved successful. Yet Dr. R. concludes his faithful report by observing that the plan recommended by Dr. Johnson (which was precisely that employed by himself) "would probably have proved destructive."

---

* Dr. Robson. Ed. Journal, No. 77.

12. *Hæmatemesis.** Vomiting of blood is always an alarming complaint, though not always dangerous. We consider the super-acetate of lead and opium, one of the best remedies both in this and hæmoptysis. Mr. Denton was called to a man who had vomited nearly two quarts of blood. Mr. D. bled him to 8 oz. ordering a cathartic of salts and digitalis; also acidulated drink. In two hours the vomiting returned, and the patient was again bled. Three grains of the superacetate with five of extract of hemlock, were given every three hours. The third dose stopped the hæmatemesis, which did not afterwards return.

---

13. *Pneumonia.*† Dr. Forbes has made some judicious remarks on the subject of pneumonia in this paper. He is surprised to find Dr. Good stating, that the inflammation in pleuritis vera commences on that side (it ought to have been portion) of the membrane which lines the ribs. Dr. Cullen and Dr. Forbes are of opinion that this is a very rare occurrence. So it has appeared in our autopsical researches also. These "ingenious divisions," however, have little influence on our practice. As Dr. Forbes observes, it is often surprising how thoracic inflammation, even of the acute kind, will terminate safely although it may have been allowed to run on unmolested for eight or ten days at first. Many recover after symptoms of effusion have actually occurred. Dr. Forbes shews the superiority of one full bleeding at the commencement of pneumonia over small and repeated evacuations. Unless there are some counter-indications, Dr. Forbes permits the blood to flow *pleno rivo*, until respiration is performed without pain, and the pulse reduced in force and frequency. The generality of Dr. Forbes' patients (soldiers) lost, at the first bleeding, from 30 to 40 ounces of blood—many from 50 to 60, and "some have borne with advantage the loss of from 60 to 70 ounces." Medical men in private practice can scarcely credit these reports, but nevertheless we know them to be true. The pulse in this, as in many other diseases, is fallacious. It is sometimes scarcely accelerated when there is severe phlogosis. A slow pulse therefore ought not to deter us from using active meaures where the other symptoms of pneumonia are present. "Blisters and digitalis, says our author, are no doubt useful occasionally, *when large bleedings are contra-indicated;* but in general I have little faith in them." We agree with him that—"the most useful auxiliary to blood-letting, in diminishing the action of the heart and arteries, is the tartarized antimony given in nauseating doses." In protracted cases, where symptoms of effusion are present, a combination of squills, digitalis, and calomel is employed with much benefit.

---

* Mr. Denton. Med. Repos. No. 119.

† Dr. Forbes. Med. and Phys. Journal, No. 291.

14. *Phthisis Pulmonalis.** Mr. Gaitskell, has here adduced an instance which apparently proves the efficacy of the tartar emetic ointment in arresting the progress of pulmonary consumption. The patient was an unmarried female, 30 years of age, and subject to catarrhal affections. In the spring of 1822 she was suddenly seized with all the symptoms of pneumonia, and was neglected in respect to treatment. In about four months after this she presented herself to Mr. G. in a state of considerable emaciation, with evening fever, morning perspirations, pulse 120, dyspnœa, constant teasing cough "with much purulent expectoration." Some blood was taken from the arm, the bowels were kept soluble with neutral salts, and the skin perspirable by the Dovar's powder. Yet the symptoms all went on increasing. About this time Dr. Jenner's pamphlet fell into our author's hands, and he applied the antimoniated ointment to the arm, between the elbow and axilla. In the course of a week a large crop of pustules appeared. As soon as the irritation of these was appeased, another crop was elicited on the other arm, during the height of which eruption the whole skin of the body became covered with numerous small itching papulæ, which deprived the patient of all comfort for more than a week. The constitutional and local symptoms now gradually abated; the pulse came down to 80; the appetite returned; and the patient perfectly recovered.

---

15. *Concussion of the Brain.*† The patient was Mr. Shipman's apprentice, who struck his head against a brass nob, but did not wound the scalp. The part stricken was the anterior edge of the right parietal bone. In the course of the day he complained of pain and heat of the part, attended with sickness and vomiting. In the evening he was delirious, with great irritability. The sense of hearing was morbidly acute. Head shaved, and 20 leeches applied, with cold evaporating lotions. Twenty ounces of blood from the arm. By these means and brisk purgatives he was relieved; but next afternoon the symptoms returned, when he was again bled, but without relief. Purgatives again mitigated the symptoms. On the third day he became blind, deaf, and dumb. He appeared hysterical. A calomel purge immediately relieved these symptoms. On the fourth morning, he again became blind, &c. and convulsed. Beween 20 and 30 leeches brought no amendment. A calomel cathartic again dissipated these phenomena, and open bowels kept him free from them till his complete recovery. During the whole period of the symptoms abovementioned, there was acute pain on pressure over the epigastric region. From this and other circumstances, we agree with the intelligent narrator of the case in believing, that the obscuration of the senses above described depended on irritation in the primæ viæ. The blow on the head might derange the

* Mr. Gaitskell, Med. Repos. 114.

† Mr. Geo. Shipman. Med. and Phys. Journal, No. 298.

chylopoietic functions, and this derangement might very readily react on the sensorium, and produce the phenomena in question.

16. *Internal Hæmorrhage.** A young gentleman, 14 years of age, was at school two days before he died, and feeling a little uneasiness in his bowels, took a dose of aperient medicine which operated, and next morning he was apparently well. He returned to school, but in the night he was seized with severe pain in the abdomen which rapidly increased, and in a few hours terminated in death. An eminent physician who was called in, ascribed the disease entirely to spasm. During the attack the countenance was pale, the pulse small and rapid; the abdomen felt extremely hard, and pressure caused some pain.

On opening the abdomen, there presented a considerable quantity of bloody serum, but no appearance of peritoneal inflammation. The greater part of the small intestines was of a dark red colour, and the mesenteric veins tinged with blood. Underneath the peritoneal coat of the small intestines there were numerous minute spots of extravasated blood. On cutting open the ileum, it was found that a large effusion of blood had taken place into its cavity—its mucous coat being of a crimson hue, and the glands enlarged, giving the internal surface of the gut, in many parts, a granular appearance. The whole of the internal surface of the diseased portion of intestine was lined with a thin coagulum of blood, " but when this was removed, and the intestine washed with warm water, *it retained its crimson colour.*" On examination, the internal coat of the lower two thirds of the jejunum was found in the same state as that of the ileum, and from it the effusion of blood had equally taken place. It was estimated that between five and six pints of blood had been extravasated.

In respect to the cause of this hæmorrhage, one gentleman present at the dissection attributed it to *congestion from spasm*—a curious cause truly! Mr. Lloyd is more disposed to place it to the account of " obstruction in some of the large venous trunks." The latter was not proved by examination, and therefore can only rank as a conjecture. For our own parts, we are not inclined to agree with either party. We consider the hæmorrhage as owing to a morbid condition of the minute vessels of the part, inducing that determination of blood which we see taking place to the membranes of the brain—to the lungs—to the stomach, and to many other structures in the human frame.

17. *Hydatid Simulating Ascites:*† Mr. Pretty, of Mabledon-place, has related a very curious case in our respected cotemporary,

* Mr. Lloyd. Med. Repos. No. 114.

† Mr. Pretty. Med. and Phys. Journal, 296.

which we witnessed ourselves, both before and after death. A midwife to an extensive charity, aged 32, had borne five children. Four years ago her illness commenced with agonizing pains in her head, which confined her nineteen months to her bed, and continued, with more or less intensity, till her death. For these pains she had been salivated, but without relief. She also became a patient in Guy's Hospital, and was discharged without benefit. An abdominal disease was now added to that of the head. When Mr. Bagster (Mr. Pretty's partner) was consulted (several months before her death) he found an enlargement of the liver—ascites (as he then supposed) and anasarca of the lower extremities, together with cough, expectoration, and difficulty of breathing. The usual remedies were tried against the dropsical affection; but failing, she was tapped in the linea alba, and about six quarts of water were drawn off, but without diminishing the size of the abdomen as much as was expected. The enlarged liver could be distinctly felt, but no other tumour. The abdomen soon filled again, and the operation was again performed, but without removing more than two pints of a greenish-coloured ropy fluid. The fluid first evacuated by paracentesis became so coagulated as scarcely to drain through the holes of a common sink; and that which came off in the second operation was still thicker. A very severe bowel complaint now came on, and she sunk early in July 1823.

*Dissection.* The edge of the liver could be distinctly traced low down in the abdomen. On throwing back the parietes, a firm white substance concealing the whole of the intestines came in view, and puzzled the operators for some time to make out what it was. When cut into longitudinally a greenish-coloured fluid escaped, and large masses of gelatinous substances were easily removed from its cavity. In short this fluid and coagula were contained in an enormous cyst, or hydatid, extending from the liver to the superior aperture of the pelvis covering the other abdominal viscera, and forming attachments to the peritoneum lining the abdomen, excepting in front, where there was a free space of a few inches in extent. It had no connexion with the ovaria or the uterus. The parietes of the cyst were about an eighth of an inch in thickness. The intestines were glued together with coagulable lymph. The liver was enormously enlarged, but not tuberculated, weighing nine pounds avoirdupois. Nothing particular in the thorax. On examining the head, an ulcer was found over the os frontis which had been open for nine or ten months. Four or five tubercles, the size and shape of a common horse-bean, were dispersed irregularly under the integuments of the head. When cut into, they discharged yellow pus, leaving the bone carious underneath. The dura mater adhered in several places with great firmness to the cranium, and was thickened.

We think it quite evident that the trochar, in both operations, had penetrated the hydatid, whence the discharge issued. In respect to the affection of the head, there is reason to suspect a syphilitic taint. All, however, that could be collected from the husband and friends was, that Mrs. N. in the course of her practice, had attended a woman, and contracted a sore on the thumb which was thought to be venereal, as she had ulcerated throat about six weeks subsequently. Salivation was employed. This occurred eleven years before her death. The case is an interesting one.

---

18. *Expulsion of a Portion of Intestine per Anum.** It is now pretty clearly ascertained that an *invaginated* portion of intestine may become sphacelated at its extremities, detached from its place, and discharged from the body, without necessarily destroying life. The following appears to be a sufficiently well authenticated case of this kind.

L. B. four years of age, of feeble constitution, but lively and healthy, was seized with small pox on the 23d July, 1820, and took no medicine till the 4th of August, when Dr. Legoupil was summoned, in consequence of the child having, for some days, complained much of violent colicky pains in the belly, accompanied by much discharge of blood per anum, but no vomiting or hiccup. Our author found the abdomen slightly distended—the right iliac region being more tender and depressed than the rest. The breathing was natural—the pulse strong, and quickened. The pustules were beginning to dry off—and there was a disagreeable putrid smell about the child's bed. The same day there appeared at the anus a purplish red tumour, the size of a pullet's egg, from the surface of which some drops of blood issued. It was supposed to be a prolapsus of the mucous membrane of the rectum, and fomentations were applied. On the fifth, the tumour exhaled a putrid odour, the stools passing in a liquid state. On the 6th, 7th, and 8th, the same state continued; but on the 9th the swelling burst forth, drawing after it a cylindrical body resembling a piece of small intestine, and about six inches in length. This substance was carefully examined by Drs. Legoupil and Delisle, and afterwards sent to the Societé de Medecine de Paris, where it was recognized to be the entire of the cæcum, and about six inches of the ileum. The child recovered and did well.

Another case, still more remarkable, has lately been presented to the Royal Academy of Medecine, by Larrey, Roux, and Beclard.† The patient, after labouring for twelve days with symptoms of internal strangulation, as obstinate constipation, fæcal vomitings, hiccup, abdominal pain, tumour in the right iliac region, &c. discharged a portion (supposed) of intestine and mesentery thirty French inches in lenth. The patient now recovered, with the exception of a painful sensation remaining in the right iliac region. He died three months afterwards of peritoneal inflammation; but leave could not be obtained to open the body.

In these cases Nature effects a junction of the sound intestinal tube, by uniting the upper and lower extremities.

---

* Journal Général de Medecine, vol. 73.

† Revue Medicale for August, 1823.

19. *Tetanus.** Mr. Harvey, of Castleheadingham, has related a case of traumatic tetanus successfully treated by opium in moderate doses. The patient was a wheelwright, who nearly cut off the end of his middle finger with an axe. About 16 days afterwards the jaw became locked, and tetanus was established. Opium, in moderate doses, with occasional purgatives, and antispasmodics, conducted to a successful issue, in about six weeks. The disease, in this instance, does not appear to have been of a violent kind. It became chronic, in which cases success generally ensues. It is, however, very creditable to the zeal and perseverance of the surgeon in attendance.

---

20. *Acute Rheumatism.*† We have several times hinted to practitioners the impropriety of attempting the reduction of acute rheumatism by the same decisive system of depletion which we use in the common phlegmasiæ of internal organs or structures. We have often seen the bad consequences of such treatment, in protracting the disease, debilitating the patient, and sometimes producing metastasis of the inflammation from exterior to interior parts. The following case (which we shall greatly abridge) is an exemplification of these remarks.

Mrs D——, aged 40, had had three or four attacks of acute rheumatism during the last ten years. On the 26th of February, she was seized with the premonitory symptoms of fever which soon evinced the rheumatic character, and by the 2d of March, when Dr. Kemper visited the patient, the general inflammatory state had acquired a great degree of intensity, and "the joints of her limbs, from the elbows and knees downwards, were affected with swelling redness, and most acute pain." Twenty-four ounces of blood drawn from the arm—a cathartic of calomel and jalap. In the evening the same venesection and purgative repeated. 3d March. Twenty-four ounces of blood drawn off—in the evening 16 ounces. 4th. Sixteen ounces, and in the evening 16 ounces more. 5th. Twice bled to 12 ounces each time. 6th. Another venesection of 12 ounces. Thus in five days about 11 pints of blood had been drawn from a woman of spare habit. Dr. K. now thought he had subdued the disease; but he was deceived. A new train of symptoms developed themselves more formidable than the inflammatory. The patient was seized with faintness, followed by darting pains through the lungs, with hurried and difficult respiration. "There now appeared to be hardly any remains of the disease externally." Laudanum, blisters, two small venesections, diaphoretics, laxatives, &c. were employed for ten days, "without any sensible alteration of the symptoms." About the 17th day, an œdematous swelling of the feet took place. Digitalis, blisters, infusion of bark, &c. gave some relief, and reduced the pulse; but the cough and difficulty of breathing still continued troublesome. On the 27th day, calomel, digitalis, and antimonial powder were

---

* Mr. G. Harvey. Med. and Phys. Journal, No. 298.
† Dr. Chapman's Journal, No. 12.

given every six hours. "Her cough was checked in a few hours, and in about two days entirely left her. The action of the kidneys, at the same time, was considerably increased—the swelling began to subside, and the pulse sank to 50 in the minute, and irregular—without any change in the respiration." A metastasis now took place to the head, and became very alarming. Fortunately the rheumatic inflammation returned to the extremities, and the interior organs were relieved. She recovered; but let her beware of another attack!

We think this case may afford a useful lesson to those practitioners who see nothing in acute rheumatism but common inflammation; and who, when they draw the lancet, throw away the scabbard, till they conquer their enemy. We can assure them that in thus conquering one enemy they will frequently set up another, and a much more formidable opponent.

---

21. *Carbuncle.** Mr. Blackadder, in his valuable treatise on hospital gangrene, has recommended the application of escharotics in cases of carbuncle. Mr. Swallow was called in by Lord Clermont for a well-characterized affection of this kind, situated on the back of the neck, over the cervical vertebræ. He had but just recovered from a similar attack under the able care of Mr. Guthrie of London. Mr. Swallow immediately made a deep crucial incision through the whole extent of the carbuncle, and filled up the incised spaces with dossils of lint well moistened in equal parts of the liq. arsenicalis and water, the fluid application being renewed every hour or two. After twenty-four hours' application, a slight eschar began to form, the surrounding inflammation and pain evidently diminishing. Twelve hours' longer continuation produced a sufficient eschar, and the pain and inflammation ceased. A common poultice was then applied frequently till the eschar separated and left a clear wound which was easily healed.

Mr. Swallow, who had charge of three large hospitals in Spain, appropriated solely for hospital gangrene, never saw any ill effects resulting from the application of arsenic in the gangrene abovementioned. The quick formation of the eschar, he believes, prevents the absorption of the deleterious substance.

---

22. *Physiological Pathology.*† A case was lately read by M. Royer-Collard, at the Royal Academy of Medicine of Paris, detailing the phenomena attendant on disease of the anterior portion of the spinal marrow. The man had been insane for more than 17 years, ten of which were past in a state of stupidity. The lower extremities were unsteady, and for some years completely paralytic—but their *sensibility* was unimpaired. On *dissection*, the pia mater covering the anterior face of the spinal marrow was of a yellowish colour—the cor-

---

* Mr. Swallow. Med. and Phys. Journal for September, 1822.

† Revue Medicale, September 1823.

pora olivaria and pyramidalia, and also the bundles composing the anterior part of the spinal chord were greyish and soft as bouillie. The *anterior roots* of the spinal nerves were in a similar state. The *posterior* portion of the spinal chord, and the *posterior roots* of the nerves were healthy. This is another confirmation of the doctrines of Bell, Magendie, &c.

---

23. *Diabetes.** Dr. Carter is publishing a series of interesting hospital reports in our respected cotemporary, of which reports we shall, from time to time, take some notice. A case is related of diabetes, in a man 33 years of age, where the disease resisted various methods of treatment till—" hard work, aided by warm clothing, and a scruple of Dover's powder at night, entirely removed the disease." An inordinate and unhealthy action of any one organ is pretty generally restrained by increasing the function of some other organ. It is evident that the skin, as an extensive outlet, sympathising powerfully with almost all the glandular viscera, is an important agent in the removal as well as in the production of diseases. Its agency, therefore, should generally be employed in diabetes. Dr. Carters' practice, he observes himself, is not new—but this is of little consequence, provided it is useful.

